# Fosfomycin: Pharmacological, Clinical and Future Perspectives

**DOI:** 10.3390/antibiotics6040024

**Published:** 2017-10-31

**Authors:** Anneke Corinne Dijkmans, Natalia Veneranda Ortiz Zacarías, Jacobus Burggraaf, Johan Willem Mouton, Erik Bert Wilms, Cees van Nieuwkoop, Daniel Johannes Touw, Jasper Stevens, Ingrid Maria Catharina Kamerling

**Affiliations:** 1 Centre for Human Drug Research, Leiden, 2333 CL, The Netherlands; annekedijkmans@hotmail.com (A.C.D.); n.v.ortiz.zacarias@lacdr.leidenuniv.nl (N.V.O.Z.); kb@chdr.nl (J.B.); j.stevens@umcg.nl (J.S.); 2 Department of Medical Microbiology, Albert Schweitzer Hospital, Dordrecht, 3318 AT, The Netherlands; 3 Department of Medical Microbiology, Radboud University Medical Center, Nijmegen, 6500 HB, The Netherlands; jwmouton@gmail.com; 4 Department of Medical Microbiology and Infectious Diseases, Erasmus Medical Center, Rotterdam, 3015 CN, The Netherlands; 5 Hospital Pharmacy, The Hague Hospitals, The Hague, 2545 AB, The Netherlands; e.wilms@ahz.nl; 6 Department of Internal Medicine, Haga Teaching Hospital, The Hague, 2566 MJ, The Netherlands; c.vannieuwkoop@hagaziekenhuis.nl; 7 Groningen Research Institute for Asthma and COPD, Department of Clinical Pharmacy and Pharmacology, University Medical Center Groningen, University of Groningen, Groningen, 9713 GZ, The Netherlands; d.j.touw@umcg.nl

**Keywords:** fosfomycin, pharmacokinetics, multidrug resistance, antimicrobial activity

## Abstract

Fosfomycin is a bactericidal, low-molecular weight, broad-spectrum antibiotic, with putative activity against several bacteria, including multidrug-resistant Gram-negative bacteria, by irreversibly inhibiting an early stage in cell wall synthesis. Evidence suggests that fosfomycin has a synergistic effect when used in combination with other antimicrobial agents that act via a different mechanism of action, thereby allowing for reduced dosages and lower toxicity. Fosfomycin does not bind to plasma proteins and is cleared via the kidneys. Due to its extensive tissue penetration, fosfomycin may be indicated for infections of the CNS, soft tissues, bone, lungs, and abscesses. The oral bioavailability of fosfomycin tromethamine is <50%; therefore, oral administration of fosfomycin tromethamine is approved only as a 3-gram one-time dose for treating urinary tract infections. However, based on published PK parameters, PK/PD simulations have been performed for several multiple-dose regimens, which might lead to the future use of fosfomycin for treating complicated infections with multidrug-resistant bacteria. Because essential pharmacological information and knowledge regarding mechanisms of resistance are currently limited and/or controversial, further studies are urgently needed, and fosfomycin monotherapy should be avoided.

## 1. Introduction

The discovery of antibiotics in the 1920s was one of the greatest breakthroughs in the history of healthcare, leading to a marked decrease in both morbidity and mortality associated with bacterial infections [1]. However, the intensive and extensive use and misuse of antibiotics over the past 50 years has contributed to the emergence and spread of antibiotic-resistant bacterial strains [2,3,4]. This increase and global spread of multidrug-resistant (MDR) bacteria is particularly alarming [3,5], and the World Health Organization has identified antibacterial drug resistance as a major threat to global public health.

The decrease in the number of effective antibiotics—together with a relative paucity of new antimicrobial drugs—is particularly relevant for treating infections with Gram-negative MDR bacteria [6,7,8]. To overcome this problem, the reassessment and reintroduction of “old” antibiotics has emerged as a viable strategy [9,10]. However, these antibiotics were never subjected to the rigorous drug development program that is currently mandatory for receiving marketing authorization. Thus, the pharmacological information needed in order to develop optimal dosing regimens with maximal activity and minimal toxicity is limited [9,11]. One such “old” antibiotic is fosfomycin, a broad-spectrum antibiotic that was originally developed more than 45 years ago. Because it has both in vitro and in vivo activity against a wide range of MDR bacteria, as well as XDR (extensively drug-resistant) and PDR (pan-drug-resistant) bacteria, fosfomycin is potentially a good candidate for treating infections with these bacteria [12,13,14,15,16,17,18].

In this review, we discuss the potential for using fosfomycin to treat MDR bacterial infections. Specifically, we review the currently available pharmacological data, with a focus on the chemistry, pharmacokinetics, pharmacodynamics, and clinical use of fosfomycin.

## 2. Methods

### 2.1. Systematic Search Strategy

The PUBMED/MEDLINE and OVID/EMBASE databases were searched systematically in February 2016 to identify all published relevant articles regarding fosfomycin. To be as comprehensive as possible, the search terms included synonyms of fosfomycin in the article titles.

The search strategies were designed and performed by a specialist librarian and were restricted to journals published in English or Dutch. No other publication or date restrictions were applied. A comprehensive database of the retrieved articles was created, and duplicate publications were removed. The abstract of each identified publication was then independently reviewed by the first author (A.C. Dijkmans) and last author (I.M.C. Kamerling). We then obtained and reviewed the full-text version of all articles that focused on multidrug-resistant Gram-negative bacteria (e.g., Enterobacteriaceae, *A. baumannii*, and *P. aeruginosa*), pharmacokinetics, pharmacodynamics, critically ill patients, treatment outcome, and/or mode of action. To search for any additional relevant articles, we screened the reference lists of the full-text articles, as well as relevant guidelines and references from the cited product information.

A final check was performed prior to submission of the manuscript in order to update the systematic search and include any new publications.

### 2.2. PUBMED/MEDLINE

PUBMED/MEDLINE was searched using the following terms: (“Fosfomycin”[Majr] OR phosphomycin[ti] OR fosfomycin[ti] OR phosphonomycin[ti] OR fosfonomycin[ti] OR monuril[ti] OR tromethamine[ti] OR trometamine[ti] OR trometamol[ti] OR tromethamol[ti]) AND (eng[la] OR dut[la]).

### 2.3. OVID/EMBASE

OVID/EMBASE was searched using the following terms: (exp *fosfomycin/ OR phosphomycin.ti. OR fosfomycin.ti. OR phosphonomycin.ti. OR fosfonomycin.ti. OR monuril.ti. OR tromethamine.ti. OR trometamine.ti. OR trometamol.ti. OR tromethamol.ti.) AND (english.lg. OR dutch.lg.).

## 3. Results

**Figure 1:**
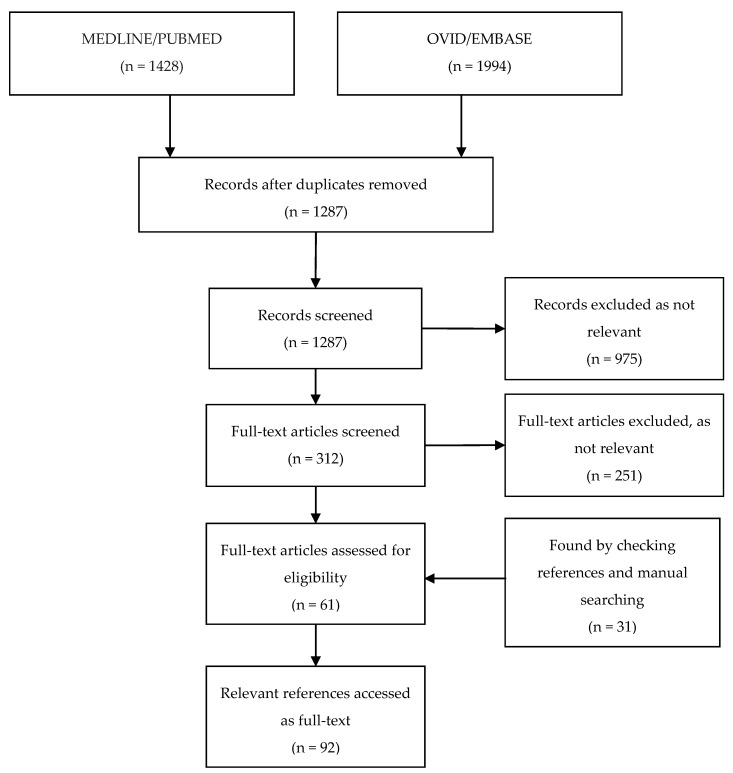
Flow-chart depicting the systematic search process and articles included.

In total, our combined search of the databases PUBMED/MEDLINE and OVID/EMBASE retrieved 3422 records; after 2135 duplicates were removed, 1287 unique publications were screened (Figure 1). Of the remaining 1287 records that were screened by title and abstract, 975 were excluded as they were judged not relevant to the topic. The remaining 312 records were examined as full-text articles, and an additional 251 were excluded, leaving 61 articles. An additional 31 articles were identified by manually checking the included publications and product information. Thus, a total of 92 articles were included in our analysis.

## 4. Pharmacology of Fosfomycin for Treating MDR Bacteria

### 4.1. Chemistry

**Figure 2:**
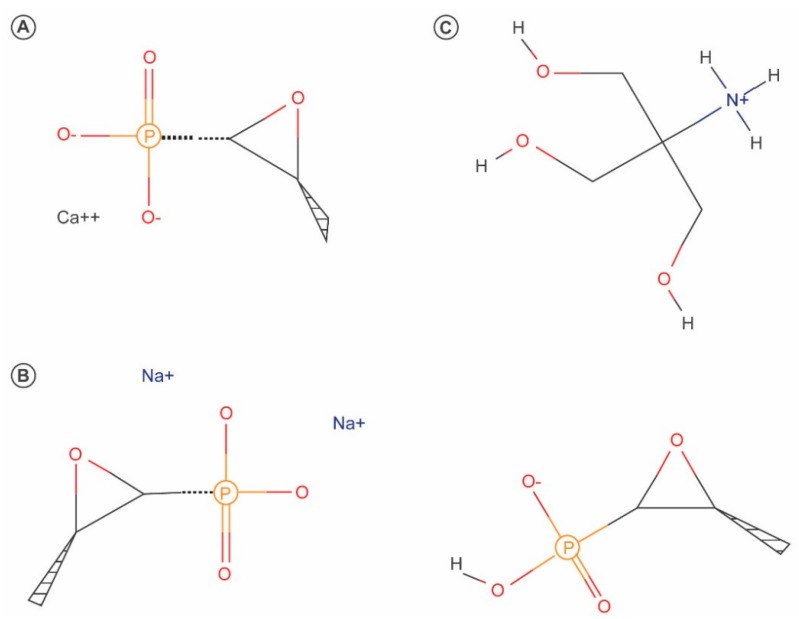
Chemical structures of fosfomycin calcium (**A**), fosfomycin disodium (**B**) and fosfomycin tromethamine (**C**).

Fosfomycin is a bactericidal broad-spectrum antibiotic first isolated in 1969 from cultures of *Streptomyces* spp. [19]. Fosfomycin, which is currently produced using a synthetic process, is a low-molecular weight (138 g/mol), highly polar phosphonic acid derivative (cis–1,2-epoxypropyl phosphonic acid) that represents its own class of antibiotics [20]. Fosfomycin was initially marketed as both a calcium salt formulation (fosfomycin calcium) for oral administration and a more hydrophilic disodium salt (fosfomycin disodium) for parenteral administration. Later, because of its improved bioavailability, fosfomycin tromethamine became the standard formulation for oral administration [20,21]. The chemical structures of the various formulations of fosfomycin are shown in Figure 2.

### 4.2. Pharmacokinetics of Fosfomycin

#### 4.2.1. Absorption

Orally administered fosfomycin is absorbed partially in the small intestine via two proposed mechanisms: (*i*) a saturable carrier-mediated system associated with a phosphate transport system, and (*ii*) a non-saturable process with first-order kinetics [22]. Studies with fosfomycin calcium have shown that before reaching the small intestine, fosfomycin undergoes acid-catalyzed hydrolysis in the stomach, where intragastric acidity and gastric emptying rate can affect the extent of fosfomycin’s hydrolytic degradation and—consequently—its bioavailability [23]. Variations between individuals with respect to intragastric acidity and gastric emptying rate may also explain the high variability in serum levels achieved after oral administration of fosfomycin [23,24].

Tromethamine is a pH-elevating (i.e., alkaline) organic compound believed to slow acid-catalyzed hydrolysis. As mentioned above, fosfomycin tromethamine is now the preferred oral formulation due to its improved properties compared to fosfomycin calcium, including higher bioavailability (F) which ranges from 33% to 44% [21,25,26] (compared to 12–37% for the calcium salt [21,27,28]). When bioavailability was calculated from urinary excretion data following oral and IV administration of fosfomycin tromethamine, values as high as 58% have been calculated [25]. Although the bioavailability of both salts is reduced when taken orally following food [24,29], when taken under fasting conditions, serum concentrations of the tromethamine salt are approximately 2–4-fold higher than the calcium formulation [21,30]. However, because no cross-over study has been performed, a systematic study of bioavailability is recommended.

Despite the improved bioavailability achieved with orally administered fosfomycin tromethamine, maximum concentrations (C_max_) of fosfomycin are still well below the C_max_ values achieved following IV administration [21,31]. For example, 2–2.5 h after a single fasting oral dose of fosfomycin tromethamine at 3 g (approximately 50 mg/kg body weight), C_max_ is 21.8–32.1 mg/L, with a total area under the serum concentration-time curve (AUC) of 145–193 mg·h/L [21,25,26]. In contrast, after IV administration of the same dose of fosfomycin disodium, C_max_ was 276–370 mg/L, with an AUC of 405–448 mg·h/L [21,25,26].

#### 4.2.2. Distribution and Tissue Penetration

Fosfomycin binds to plasma proteins at only negligible levels [31] and is distributed widely into a variety of tissues; in addition to serum, biologically relevant concentrations of fosfomycin have been measured in the kidneys, bladder, prostate, lungs, bone, and cerebrospinal fluid, as well as in inflamed tissues and abscess fluid [32,33,34,35,36,37,38,39,40].

The apparent volume of distribution (V_d_/F) following oral administration of fosfomycin tromethamine is approximately 100–170 L for a 70-kg individual [29,30]. In contrast, because of its higher bioavailability, IV-administered fosfomycin disodium has a reported V_d_ of 9–30 L at steady state, and values of 3–12 L have been reported for both the central (Vc) and peripheral (Vp) compartments [25,27,28,32,36,41,42].

#### 4.2.3. Metabolism and Excretion

Approximately 90% of an IV dose of 3 g fosfomycin disodium is recovered unchanged in the urine 36–48 h after dosing [21,25,26]. In contrast, only 40–50% of a 3 g oral dose of fosfomycin disodium is recovered; this difference compared to an IV dose is due primarily to incomplete absorption of oral fosfomycin disodium [21,25,26,29]. Following an oral dose of fosfomycin tromethamine, approximately 10% of the original dose is recovered unchanged in the feces [29].

Segre et al. reported that the fraction of the original dose excreted in the urine decreases as the oral dose increases [25], suggesting decreased absorption at higher doses. However, their study used a relatively limited range of doses (2, 3, and 4 g) in a small number of individuals (*n* = 12). On the other hand, urinary concentrations >128 mg/L are maintained 24–48 h after an oral dose of 2, 3, or 4 g and 12–24 h after an IV dose of 3 g [26].

In general, the total clearance rate ranges from 5 to 10 L/h, whereas renal clearance ranges from 6 to 8 L/h [25,27,31,32,35,36,41,43]. Fosfomycin has also been detected in the bile, with biliary concentrations of approximately 20% of the serum concentration [31,44,45]. Given this finding, Segre et al. suggested that fosfomycin undergoes biliary recirculation, based on the presence of secondary peaks in serum drug concentration following oral administration and based on the concentrations of fosfomycin measured in the bile [25,31,38,44,45].

In healthy individuals, IV fosfomycin is distributed in and eliminated from the serum in a bi-exponential manner; the serum disposition half-life (t_1/2α_) of fosfomycin is 0.18–0.38 h [28,43], and the terminal (or elimination) half-life (t_1/2β_) of fosfomycin is 1.9–3.9 h [21,25,26,27,28,32,35,36,43]. In contrast, the t_1/2β_ is longer following an oral dose of fosfomycin tromethamine (3.6–8.28 h [21,26,30]), which can be explained by a longer absorption phase. In patients who have renal failure and/or are receiving hemodialysis, the t_1/2β_ of fosfomycin can be as long as 50 h, depending on the level of renal function; therefore, the dosing schedule should be adjusted accordingly, particularly if creatinine clearance (CL_CR_) drops below 40 mL/min [43,44,46].

An overview of the farmacokinetics is given in Table 1.

### 4.3. Pharmacodynamics of Fosfomycin

#### 4.3.1. Mechanism of Action

In general, antibiotics exert their bactericidal or bacteriostatic activity by targeting the microorganism’s essential physiological and/or metabolic functions, including protein, DNA, RNA, or cell wall synthesis and cell membrane organization. Fosfomycin has a unique mechanism of action in which it irreversibly inhibits an early stage of bacterial cell wall biosynthesis.

**Figure 3:**
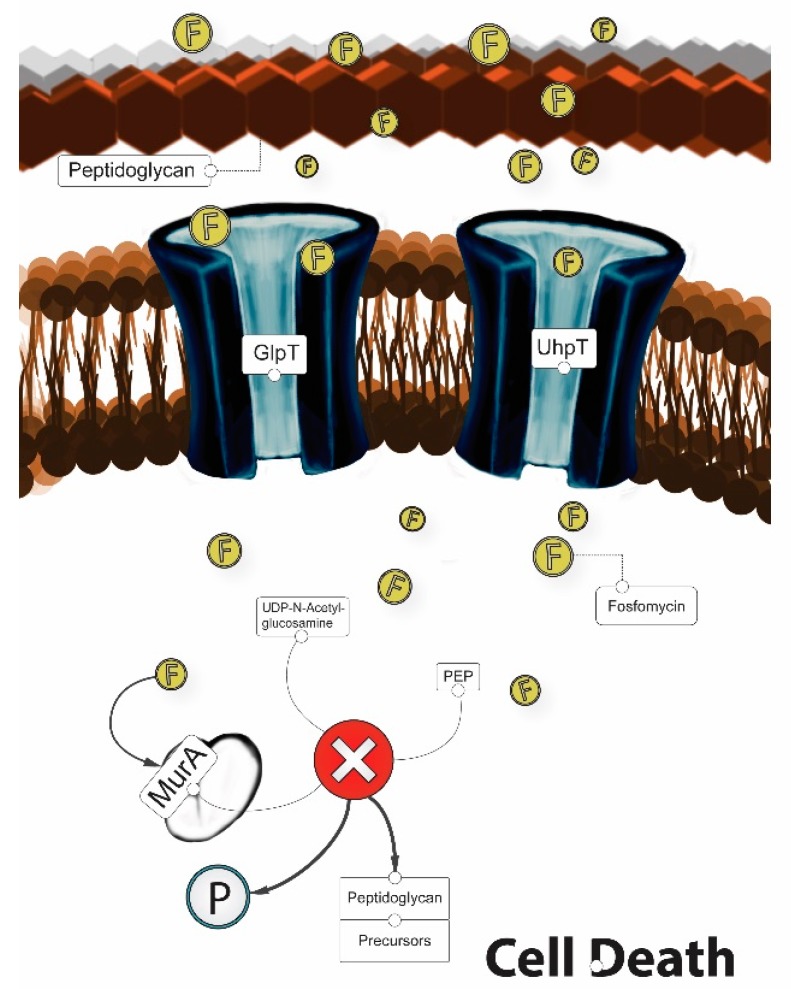
Mechanism of action of fosfomycin (“F”).

In order to exert its bactericidal activity, fosfomycin must reach the bacterial cytoplasm. To enter the cell, fosfomycin uses the active transport proteins GlpT and UhpT by mimicking both glucose-6-P (G6P) and glycerol-3-P (G3P). Thus, fosfomycin can be imported into the bacterial cell via the hexose monophosphate transport system (which is induced by G6P) and via the L-a-glycerophosphate transport system (which is induced by G3P) [20,47]. Once in the cytoplasm, fosfomycin acts as an analog of phosphoenolpyruvate (PEP) and binds MurA (UDP-GlcNAc enopyruvyl transferase), thereby inactivating the enzyme enolpyruvyl transferase, an essential enzyme in peptidoglycan biosynthesis [48]. Thus, fosfomycin prevents the formation of UDP-GlcNac-3-O-enolpyruvate from UDP-GlcNAc and PEP during the first step in peptidoglycan biosynthesis, thereby leading to bacterial cell lysis and death (Figure 3) [47]. In addition, fosfomycin also decreases penicillin-binding proteins [49].

#### 4.3.2. Antibacterial Activity

Because both Gram-negative and Gram-positive bacteria require *N*-acetylmuramic acid for cell wall synthesis, fosfomycin is as a broad-spectrum antibiotic with activity against a wide range of bacteria, including *Escherichia coli*, *Proteus mirabilis*, *Klebsiella pneumoniae*, *Enterobacter* spp., *Citrobacter* spp., and *Salmonella typhi* [12,20,50,51,52]. However, due to a paucity of preclinical and clinical data, no universally accepted minimum inhibitory concentration (MIC) values have been defined for the susceptibility and resistance to fosfomycin; overall, the MIC for susceptibility ranges from ≤32 to ≤64 mg/L, and the MIC for resistance ranges from >32 to >256 mg/L, according to the Clinical and Laboratory Standards Institute (CLSI) and the European Committee on Antimicrobial Susceptibility Testing (EUCAST) [14,53].

Several studies have investigated the microbiological activity and efficacy of fosfomycin against several MDR, XDR, and PDR strains of Gram-negative bacteria. In this respect, fosfomycin has been reported to have both in vitro and in vivo activity against several MDR and XDR species of Enterobacteriaceae, including species that express extended-spectrum β-lactamases (ESBL) and metallo-β-lactamases (MBL) [14,15,16,17,18]. Due to the broad range of MIC values and differences in methods used to test susceptibility (e.g., agar dilution, microdilution, E-test), it is difficult to compare the results of different studies. However, given that some studies found that more than 90% of MDR and XDR isolates are susceptible to fosfomycin, fosfomycin is a promising candidate for treating infections with these pathogens [15,16], provided that in vivo results support the in vitro data.

MDR *P. aeruginosa* and *A. baumannii* are Gram-negative pathogens primarily responsible for nosocomial (i.e., hospital-acquired) infections, particularly in intensive care units [54]. A systematic review of microbiological, animal, and clinical studies using non-fermenting Gram-negative bacilli concluded that using fosfomycin in combined therapy may provide a safe and effective therapeutic option for treating infections due to MDR *P. aeruginosa* [13]. The clinical efficacy of fosfomycin against MDR-bacteria, including *P. aeruginosa*, has been suggested in patients with severe infections and critical conditions [18], and in cystic fibrosis patients with infective pulmonary exacerbations [55,56]. However, when used as monotherapy, *P. aeruginosa* should generally be regarded resistant to fosfomycin [57] and its use in *P. aeruginosa* infections should ideally be reversed for additional evaluation in clinical studies because the increased bacterial killing of combination therapy does not prevent the emergence of fosfomycin resistance [58]. In contrast, nearly all isolates of *A. baumannii* are resistant to fosfomycin, with a MIC_90_ value higher than 512 mg/L and there are no data on its use in combination therapy [14].

## 5. Fosfomycin Resistance

Three separate mechanisms of fosfomycin resistance have been reported [59]. The first mechanism is based on decreased uptake by the bacterium due to mutations in the genes that encode the glycerol-3-phosphate transporter or the glucose-6-phosphate transporter [47,60,61]. The second mechanism is based on point mutations in the binding site of the targeted enzyme (MurA) [62], and several isolates of *E. coli* have clinical resistance levels (32 mg/L) due to increased expression of the *murA* gene [63].The third mechanism of resistance is based on the inactivation of fosfomycin either by enzymatic cleavage of the epoxide ring or by phosphorylation of the phosphonate group. In the presence of the metalloenzymes FosA, FosB, and FosX, the epoxide structure is cleaved, with glutathione (FosA), bacillithiol and other thiols (FosB), or water (FosX) serving as the nucleophile [64]. With respect to the phosphorylation of the phosphonate group, FomA and FomB are kinases that catalyze the phosphorylation of fosfomycin to the diphosphate and triphosphate states, respectively [65,66]. Fosfomycin dosing regimens that include a total daily dose of up to 24 g per day resulted in the emergence of a resistant subpopulation within 30–40 h of drug exposure, suggesting that resistance can occur rapidly.

### 5.1. In Vitro Synergy between Fosfomycin and Other Antibiotics

The use of combined antimicrobial therapy is recommended in specific patient populations and indications, including critically ill patients who are at high risk for developing an MDR bacterial infection and patients with a *P. aeruginosa* infection [11,67,68]. In this regard, fosfomycin has an in vitro synergistic effect of up to 100% when combined with other antimicrobial agents [69].

The synergistic effect between fosfomycin and β-lactam antibiotics is proposed to arise from the inhibition of cell wall synthesis at separate steps; fosfomycin inhibits the first enzymatic step, whereas β-lactam antibiotics inhibit the final stage in the cell wall synthesis process [70]. In addition, fosfomycin may modify the activity of penicillin-binding proteins, which may account for the synergistic effect between fosfomycin and β-lactam antibiotics [49,71,72]. Another study found that the synergistic effect between fosfomycin and ciprofloxacin is due to ciprofloxacin-mediated damage to the outer membrane, which increases the penetration and activity of fosfomycin [73]. With respect to *P. aeruginosa*, several in vitro studies found synergy between fosfomycin and a variety of other antibiotics, including aztreonam, cefepime, meropenem, imipenem, ceftazidime, gentamycin, amikacin, ciprofloxacin, and others [70,74,75]. In addition, a few studies measured the synergistic effect of combining fosfomycin with amikacin or sulbactam against *A. baumannii* strains, providing evidence that these drugs might provide an effective combination therapy for infections with this pathogen [76,77]. Fosfomycin also has synergistic effects when combined with other antibiotics for treating methicillin-resistant *S. aureus*, *Streptococcus*, *Enterococcus*, and Enterobacteriaceae species [69,70]. In addition to increasing antibacterial efficacy, fosfomycin can also reduce toxicity associated with other antibiotics such as aminoglycosides, glycopeptides, and polymyxin B, as lower doses of these drugs can be prescribed [78,79,80].

### 5.2. Properties of Fosfomycin

The reintroduction of “old” antimicrobial agents to treat MDR bacteria requires optimization of the dosing regimen. This optimization includes obtaining a thorough understanding of the drug’s pharmacokinetic (PK) and pharmacodynamic (PD) properties, thereby providing maximal antibacterial activity while minimizing toxicity and the development of resistance [11]. However, some “old” antibiotics, including fosfomycin, are currently used clinically despite uncertainty regarding the required and/or optimal exposure [11]. Therefore, it is essential to determine a rational dosing regimen based on the drug’s PK/PD properties when introduced as a therapy against MDR bacteria.

### 5.3. PK/PD Properties

Because the exposure-response relationship can differ between antibiotics, it is important to define the correct PK/PD index for each antibiotic in order to establish the PK/PD target value that will maximize clinical efficacy [11,81,82]. With respect to antimicrobials, three PK/PD indices are commonly used: T_>MIC_, which is the duration of time in which the drug concentration remains above the MIC during a dose interval; C_max_/MIC, which is the drug’s C_max_ divided by the MIC; and AUC/MIC, which is the AUC measured over a 24-h period divided by the MIC.

Relatively few in vitro studies have been performed to characterize fosfomycin’s PK/PD properties. Some such studies suggest that fosfomycin has a time-dependent bactericidal activity, specifically against the Gram-positive *S. aureus* and *S. pyogenes* strains [32,35]; therefore, based on these results T_>MIC_ should be optimized. However, in vitro studies by Mazzei et al. [83] and VanScoy et al. [84] suggest that fosfomycin shows a tendency towards a concentration-dependent bactericidal activity against *E.coli* and *P. mirabilis* strain, achieving complete sterilization at concentrations ≥4X MIC and ≥8X MIC, respectively. Moreover, an in vitro concentration-dependent post-antibiotic effect (PAE) was observed for both *E.coli* and *P. mirabilis* 3.2–3.4 h at 0.25X MIC and 3.5–4.7 h at 8X MIC [83]. However, with respect to these studies, it is not clear whether the bactericidal activity is concentration-dependent and/or time-dependent [85]. These studies however, do not provide conclusive data on the concentration- or time depending nature of bactericidal activity. Therefore, the target PK/PD to achieve during therapy remains unknown, which is a major hurdle that must be overcome in order to optimize therapy.

### 5.4. Current Clinical Indications for Fosfomycin and Potential Future Applications

#### 5.4.1. Intravenous Administration

Fosfomycin disodium is currently available in only a few European countries—namely, Spain, France, Germany, the United Kingdom, the Netherlands, Austria, and Greece—where it is approved for the treatment of soft-tissue infection and sepsis. A Fosfomycin disodium adult dose of 12–24 g daily is commonly administered in 2–4 separate infusions [51].

Due to is extensive tissue penetration, fosfomycin has emerged as a potential therapy for treating infections in the central nervous system (CNS) [32], soft tissues [33,39,40], bone [39], lungs [34], and abscesses [36]. Fosfomycin has high penetration into the interstitial fluid of soft tissues [40], reaching 50–70% of the levels measured in plasma, reaching sufficiently high levels to eliminate relevant pathogens [33,40]. Moreover, Schintler et al. reported that fosfomycin might also be effective in treating “deep” infections involving the osseous matrix [39].

With respect to CNS infections, Pfausler et al. reported that three daily IV doses of 8 g provided a steady-state concentration of 16 mg/L in the cerebrospinal fluid (CSF) for more than 90% of the interval between doses [32]. Moreover, the concentration of fosfomycin in the CSF can increase by nearly threefold with meningeal inflammation [86]. With respect to suppurative lesions, Sauermann et al. reported that repeated doses of IV fosfomycin can yield a concentration of 32 mg/L fosfomycin in the abscess, albeit with high inter-individual variability in the PK of fosfomycin in the abscess fluid [36,41].

MDR bacteria such as ESBL-producing bacteria and carbapenem-resistant bacteria are still susceptible to fosfomycin [17,18], and fosfomycin is used in combination therapy for treating these infections.

The repurposing of fosfomycin based on its activity against MDR Enterobacteriaceae is an important strategy for addressing the ever-present threat of antimicrobial resistance. The AUC/MIC seems to be the dynamically linked index for determining resistance suppression. In this respect, it is essential to develop optimal dosing strategies for each MDR Enterobacteriaceae species based on PK/PD data; moreover, additional dosing regimens may need to be developed for targeting different tissue sites of infection in order to prevent the development of resistance. Another promising approach is the use of combination therapy; for example, combining fosfomycin and meropenem yielded a significant synergistic effect, but also yielded a significantly additive effect in the fosfomycin-resistant subpopulation [87].

Currently, the FOREST study group is comparing the efficacy of combining fosfomycin with meropenem in treating urinary tract infections (UTIs) with ESBL-producing *E. coli* [88].

#### 5.4.2. Oral Administration

Fosfomycin tromethamine is currently approved for use in several European countries and is only approved as a single 3-g dose for treating uncomplicated UTIs in women, specifically UTIs due to *E. coli* infection [29]. Fosfomycin tromethamine has also been investigated as a potential therapy for surgical prophylaxis in order to prevent prostate infection and even as a treatment for prostatitis due to MDR Gram-negative bacteria [37]. The use of a multiple-dose regimen with fosfomycin tromethamine has emerged as a potential strategy for treating of complicated and/or recurrent UTI, as well as infections due to MDR bacteria [89,90,91]. In this respect, simulations of the urinary concentrations of fosfomycin have been developed in order to determine the optimum dosing regimen that can provide a urinary concentration above the MIC (i.e., >16 mg/L) for seven days [89]; these simulations suggest that a single dose of 3 g administered every 72 h is sufficient to achieve the appropriate concentration. In addition, an uncontrolled, open-label, multicenter study conducted in China found that a regimen of single 3-g doses of fosfomycin tromethamine administered at two-day intervals might provide a safe, effective, and well-tolerated option for treating recurrent and/or complicated lower UTIs [90]. Thus, although the currently approved 3-g single dose of fosfomycin tromethamine is sufficient to reach efficacious concentrations in the urine, it might not be sufficient to achieve serum and/or tissue concentrations that are relevant for a clinical cure. A multiple-dose regimen of fosfomycin tromethamine might therefore be warranted for the oral treatment of more severe infections.

Ortiz et al. conducted simulations of several multiple-dose regimens using a wide range of daily doses of fosfomycin tromethamine and fosfomycin disodium [92]. The authors calculated PK/PD indices, including C_max_/MIC, AUC/MIC, and %T_>MIC_, for each dosing regimen using a MIC of 8 mg/L. They concluded that a total daily dose of 6–12 g for microorganisms with a MIC of 8 mg/L well exceeds the currently approved single dose of 3 g. However, the safety and tolerability of fosfomycin tromethamine at such high doses has not been investigated. Nevertheless, further studies are urgently needed in order to assess the PK, safety, tolerability, and efficacy of fosfomycin in both multiple-dose regimens and synergistic combinations.

## 6. Conclusions

The World Health Organization currently recognizes that antibacterial drug resistance is one of the major threats facing global public health, particularly given the reduction in the number of effective antibiotics. In this respect, reassessing and reevaluating “old” antibiotics such as fosfomycin has been proposed as a possible strategy in treating drug-resistant bacterial infections. Fosfomycin is a broad-spectrum antibiotic with both in vivo and in vitro activity against a wide range of bacteria, including MDR, XDR, and PDR bacteria. Thanks to its high tissue penetration, fosfomycin may be used in a broad range of tissues and targets, including the CNS, soft tissue, bone, lungs, and abscess fluid. Oral fosfomycin in a multiple-dose regimen has emerged as a potential strategy for treating complicated UTIs and prostatitis; however, given the relative lack of essential information regarding the pharmacological properties and mechanisms of resistance, additional studies are urgently needed. In the meantime, using fosfomycin as a monotherapy should be avoided due to the rapid development of resistance in vitro.

## Figures and Tables

**Table 1:** Overview of the reported pharmacokinetic properties of fosfomycin calcium, fosfomycin tromethamine, and fosfomycin disodium.

Ref	Dose	Study Group (N)	T_max_ (h)	t_1/2β_ (h)	V_d_ (L)	CL (L/h)	CL_R_	F (%)	k_a_	k_el_	Q
Fosfomycin calcium											
Cadorniga et al., 1977 [28]	500 mg	HV (6)	2–2.5	2.04	20.7	ND	ND	37	ND	0.12	NA
Goto et al., 1981 [27]	20 mg/kg	HV (7)	2.3 (0.3)	3.01 (0.67) ^g^	30.1 (4.6)	7.1 (1.5)	ND	28 (7.0)	1.03 (0.38)	0.24 (0.05)	NA
	40 mg/kg	HV (7)	2.7 (0.2)	5.05 (0.81) ^g^	60.2 (17.4)	9.0 (1.7)	ND	28 (8.0)	0.92 (0.40)	0.14 (0.02)	NA
Borsa et al., 1988 [30]	40 mg/kg SD	Young HV (5)	1.41 (0.67)	4.81 (1.90) ^g^	435.0 (144.0)	59.3 (23.3) ^a^	5.0 (1.1) ^a^	ND	ND	0.170 (0.084)	NA
		Elderly HV (8)	2.58 (0.54)	11.80 (6.86) ^g^	409.4 (100.4)	33.4 (23.1) ^a^	3.3 (1.1) ^a^	ND	ND	0.082 (0.047)	NA
Bergan et al., 1990 [21]	50 mg/kg	HV (8)	2.9 (0.6)	5.6 (1.8) ^g^	ND	ND	ND	12.0 (7.5)	ND	0.135 (0.053)	NA
Fosfomycin tromethamine											
Segre et al., 1987 [25]	50 mg/kg	HV (5)	2.2 (0.44)	2.43 (0.31)	10.4 (1.5)	8.3 (1.6)	7.0 (0.9)	0.44 (0.09) 0.58 (0.04) ^e^	Transit model k_10_: 1.24 (0.55) k_12_: 1.69 (0.62) k_23_: 0.34 (0.10)	k_35_: 0.69 (0.07) ^f^	NA
Borsa et al., 1988 [30]	25 mg/kg SD	Young HV (5)	1.61 (0.23)	5.37 (2.56) ^g^	186.3 (129.4)	19.4 (8.4) ^a^	10.8 (1.5) ^a^	ND	ND	0.156 (0.073)	NA
		Elderly HV (8)	2.16 (0.72)	8.28 (5.51) ^g^	101.1 (61.2)	9.7 (4.2) ^a^	2.9 (1.0) ^a^	ND	ND	0.124 (0.078)	NA
Bergan et al., 1990 [21]	25 mg/kg	HV (8)	2.6 (0.5)	3.9 (0.65) ^g^	ND	ND	ND	ND	ND	0.183 (0.031)	NA
	50 mg/kg	HV (8)	2.5 (0.8)	3.6 (0.44) ^g^	ND	ND	ND	40.6 (17.9)	ND	0.197 (0.024)	NA
Bergan et al., 1993 [26]	2 g	HV (12)	2.2 (0.9)	4.1 (0.8) ^g^	ND	ND	ND	ND	ND	0.17b	NA
	3 g	HV (12)	2.0 (0.6)	4.5 (2.1) ^g^	ND	ND	ND	32.9 (7.9)	ND	0.15b	NA
	4 g	HV (12)	2.0 (0.0)	3.9 (0.7) ^g^	ND	ND	ND	ND	ND	0.18b	NA
Fosfomycin disodium											
Kwan et al., 1971 [42]	250 or 500 mg, 10-min infusion, Single dose 500 mg every 6 h, 8 times.	HV (17)	NA	1.1 ^c^	V_c_: 12.9	7.5	7.1	NA	NA	K13: 0.62	12.4 ^b^ k_12_: 0.96 k_21_: 1.19
Cadorniga et al., 1977 [28]	500 mg, 5-min infusion	HV (6)	NA	t_1/2α_: 0.38 t_1/2β_: 2.04	V_c_: 12.9 V_p_: 7.8 V_dss_: 20.7	ND	ND	NA	NA	K13: 0.67	6.9 ^b^ k_12_: 0.54 k_21_: 0.88
Goto et al., 1981 [27]	20 mg/kg, 5-min infusion	HV (7)	NA	2.25 (0.74)	V_c_: 8.7 (2.9) V_p_: 9.8 (1.7) V_dss_: 18.5 (4.6)	7.2 (1.6)	6.0 (2.2)	NA	NA	β: 0.34 (0.12) k_10_: 0.92 (0.31)	14.2 ^b^ k_12_: 1.62 (0.76) k_21_: 1.45 (0.75)
	40 mg/kg, 5-min infusion	HV (7)	NA	2.22 (0.46)	V_c_: 8.7 (2.9) V_p_: 12.7 (2.9) V_dss_: 20.8 (3.5)	8.0 (0.8)	6.6 (0.9)	NA	NA	β: 0.32 (0.06) k_10_: 0.99 (0.22)	16.2 ^b^ k_12_: 1.84 (0.85) k_21_: 1.30 (0.49)
Lastra et al., 1983 [43]	30 mg/kg	Patients with normal renal function (9)	NA	t_1/2α_: 0.18 (0.09) t_1/2β_:1.91 (0.50)	21.2 (10.4)	7.9 (3.2)	ND	NA	NA	k_13_: 1.91 (1.29)	k_12_: 2.22 (1.49) k_21_: 1.18 (0.68)
		Patients with impaired renal function (8)	NA	t_1/2α_: 0.61 (0.18) t_1/2β_: 16.3 (11.9)	17.8 (6.8)	1.1 (0.8)	ND	NA	NA	k_13_: 0.21 (0.17)	k_12_: 0.66 (0.38) k_21_: 0.43 (0.13)
Segre et al., 1987 [25]	50 mg/kg, Single injection	HV (5)	NA	2.43 (0.31)	10.4 (1.5)	8.3 (1.6)	7.0 (0.9)	NA	NA	k_35_: 0.69 (0.07) ^f^	10.6 ^b^ k_34_: 1.00 (0.92) k_43_: 1.40 (0.91)
Bergan et al., 1990 [21]	50 mg/kg, 5-min infusion	HV (8)	NA	3.4 (1.1)	ND	ND	ND	NA	NA	0.206 (0.048)	ND
Bergan et al., 1993 [26]	3 g	HV (12)	0.02 (0.0)	2.1 (0.1)	ND	ND	ND	NA	NA	0.33 ^b^	ND
Joukhadar et al., 2003 [35]	8 g, 20-min infusion	Critically ill patients (9)	0.4 (0.1)	3.9 (0.9)	31.5 (4.5)	7.2 (1.3)	ND	NA	NA	0.18 ^b^	ND
Pfausler et al., 2004 [32]	8 g, 30-min infusion, Single dose	Patients requiring extraventricular drainage (6)	1.2 (0.4)	3.0 (1.0)	30.8 (10.2)	7.4 (2.3)	ND	NA	NA	ND	ND
	8 g, 30-min infusion, every 8 h for 5 days	Patients requiring EVD	1.5 (1.2)	4.0 (0.5)	26.3 (9.7)	5.0 (2.0)	ND	NA	NA	ND	ND
Sauermann et al., 2005 [36]	8 g, 30-min infusion, Single dose	Patients (12)	0.47 (0.12)	3.7 (2.2)	V_c_: 15.5 (4.5) V_dss_: 28.6 (9.9)	7.6 (4.1)	ND	NA	NA	0.19 ^b^	ND
Kjellsson et al., 2009 [41]	8 g, 30-min infusion, Single dose	Patients (12)	NA	1.2^c^	V_c_: 10.1 (5.4–14.8) V_p_: 9.80 (5.7–13.9)	5.8 (3.8–7.8)	ND	NA	NA	0.58 ^d^	15.4 (9.1–21.6)

HV, healthy volunteers; N, number of subjects; V_d_, apparent volume of distribution (unless specified as another reported volume); CL_R_, renal clearance; F, bioavailability; k_a_, apparent first-order absorption rate constant; k_el_, apparent first-order elimination rate constant; Q, intercompartmental clearance. ^a^ Calculated in L/h per 1.73 m^2^, ^b^ Calculated from kel and k_12_, k_21_. Q = k_12_*V1 and Q = k_21_*V2, ^c^ Calculated using the equation t_1/2_ = 0.693/kel, ^d^ Calculated from CL and central V_d_ the equations Kel = CL/Vc and Kel = 0.693/t_1/2_, ^e^ Bioavailability calculated using the PK model (F = k_12_/(k_12_+k10)) and the ratio of the amount excreted in the urine after oral and IV administration, ^f^ Rate of elimination in the urine, ^g^ Apparent terminal half-life.
